# Jottings

**Published:** 1909-12-18

**Authors:** 


### Jottings.

The Queen has sent a present of game to the Chelsea Hospital for Women.

The Cheshire County Council has decided to extend the County Asylum at a cost of £82,000.

The annual sale in aid of the Victoria Hospital for Children was held on December 1 at 1 Onslow Gardens.

Miss Carl Bennett gave an entertainment to the in-patients of the Cancer Hospital, Fulham Road, on Monday last.

The Duke of Newcastle has presented a new *x*-ray apparatus to the Queen's Hospital for Children, Hackney Road, to replace the installation given by him in 1893.

As the result of the bazaar held at Camelford House, W., in aid of the General Lying-in Hospital, York Road, S.E., it is expected that a sum of £11,000 will be handed over to the charity.

Among the exhibits at the Art Loan Exhibition, in aid of the West Norfolk and Lynn Hospital, at the St. James' Hall, King's Lynn, is a number of sepia drawings by Simpson, illustrating the King's tour in India in 1875-76, which has been graciously lent by his Majesty.

The list day of the annual sale of patients' work at the British Home and Hospital for Incurables, Crown Lane, Streatham, was opened on November 24 by the Baroness von Eckhardstein. Many of the articles for sale had been made by the inmates, and the whole of the proceeds will be for their personal use.

Lord Provost McInnes Shaw, presiding over the annual meeting of contributors to the Victoria Infirmary, Glasgow, on December 2, said that in connection with the Royal, Western, and Victorian Infirmaries some scheme of joint management might not be impractical. From the standpoint of economy he thought amalgamation would introduce improvement.

The Royal University of Ireland, we understand, will be dissolved before the end of 1909. By the provisions of the Irish Universities Act, 1908, two new Universities will be constituted—namely, the National University of Ireland and the Queen's University of Belfast. For the sake of avoiding confusion, it may be well to state the old and the new names side by side. What was the Royal University of Ireland becomes the National University of Ireland. What was Queen's College, Belfast, becomes Queen's University of Belfast.

An operating theatre and a verandah, which also serves as a fire escape, were opened on December 3 at the Hartlepool Hospital. These, together with the fact that Mr. W. C. Gray, High Sheriff of the county, has defrayed the cost of installing a new ventilating system and a new heating apparatus, form a fine scheme of improvement.

The annual sale of work in aid of the Royal National Orthopædic Hospital was opened on November 24 by Lady Lansdowne at the hospital. Lord Denbigh, chairman of the Hospital, said that when the hospital was opened by the King and Queen last July only 40 beds were occupied; the number now was 130. The question of finance was a very serions one, and they were confronted with the large debt of £25,000, which was still owing on the building fund.

A dinner in aid of the Great Northern Central Hospital was held at the Hôtel Métropole on November 24. Sir Edgar Speyer presided. The Chairman said that, including an overdraft on the building fund, the total indebtedness to their bankers amounted to £14,462. For the year 1909 they were about £3,000 short. Donations and subscriptions were announced amounting to £6,363, including £500 from the Chairman.

The additions to the North Lonsdale Hospital, comprising sitting-rooms, bedrooms, and other conveniences for the nursing staff, have been completed, and are now open for public inspection. The extension is a great improvement. Hitherto sleeping accommodation for some of the nurses has had to be found outside the hospital. This, of course, was not a satisfactory arrangement, and thanks to the Hospital Demonstration it has been possible to remedy this state of affairs.

At a meeting of the Rugeley District Hospital on Wednesday, October 13, the following resolution was carried unanimously: "That the hospital be improved by providing an operating theatre and extra wards if possible, at a minimum cost of £500, and that the Hospital Committee be requested to call into being a special committee to raise the necessary money." The passing of this resolution has interest as being the *modus vinendi* arrived at by which the party (of which the hospital's chairman, Lord Lichfield, was one), opposed to the expenditure of further money, and the surgeons who required a new operating theatre have combined their views.

